# Etiology and perinatal outcomes between early and late-onset nonimmune hydrops fetalis

**DOI:** 10.1590/1806-9282.20231723

**Published:** 2024-07-19

**Authors:** Seval Yılmaz Ergani, Müjde Can İbanoğlu, Ayberk Çakır, Çağlayan Ateş, Gökcen Örgül, Nazan Vanlı Tonyalı, Özge Yücel Çelik, Dilek Şahin

**Affiliations:** 1 Etlik Zubeyde Hanım Women's Health Training and Research Hospital, Department of Perinatology – Ankara, Turkey.; 2 Etlik Zubeyde Hanım Women's Health Training and Research Hospital, Department of Obstetrics and Gynecology – Ankara, Turkey.

**Keywords:** Cardiovascular abnormalities, Congenital abnormalities, Hydrops fetalis, Non-immune hydrops fetalis

## Abstract

**OBJECTIVE:**

We aimed to compare the etiology and perinatal outcomes of non-immune hydrops fetalis diagnosed early- and late-onset at our hospital.

**METHODS:**

The records of the patients who applied to our department were reviewed, and we reached 42 non-immune hydrops fetalis cases retrospectively and examined the medical records. Hydrops diagnosis week, birth week, accompanying anomalies, and perinatal outcomes were compared as ≤12 weeks (early-onset) and >12 weeks (late-onset).

**RESULTS:**

The prevalence of non-immune hydrops fetalis was 0.05%, and the median week of diagnosis for hydrops was 18 weeks. Consanguinity (16.7%) was found in seven pregnancies, and the other seven patients (16.7%) had a history of hydrops in previous pregnancies. Anomalies of the skeletal system, central nervous system, and gastrointestinal tract accounted for 66.7% of ≤12 weeks in non-immune hydrops fetalis cases. Cardiac abnormalities were more common (26.7%) in patients at > 12 weeks (p=0.078). A statistically significant difference was found between the distribution of week of birth and week of diagnosis (p=0.029). Notably, 66.7% of patients diagnosed before week 12 and 23.3% of patients diagnosed after week 12 delivered their babies before week 24. Spontaneous intrauterine death occurred before week 12 in 45.5% (n=5) of non-immune hydrops fetalis and after week 12 in 39.1% (n=9) of non-immune hydrops fetalis. Notably, 69.2% (n=9) of the patients who had prenatal invasive testing resulted in normal karyotype.

**CONCLUSION:**

In this study, most of the fetuses diagnosed with early-onset non-immune hydrops fetalis were born in the first 24 weeks. Additionally, live birth rates and cardiac anomalies were observed to be higher in late-onset non-immune hydrops fetalis.

## INTRODUCTION

Hydrops fetalis is defined as abnormal fluid accumulation in two fetal compartments, including fetal pleura, pericardium, intra-abdominal, and subcutaneous tissues^1^. Immune hydrops fetalis is characterized by fluid accumulation in the fetus due to erythrocyte destruction and anemia resulting from parental blood incompatibility. Hydrops cases that are not due to blood group incompatibility are referred to as nonimmune hydrops fetalis (NIHF). The prevalence of immune hydrops fetalis has decreased significantly due to the widespread use of anti-D immunoglobulin prophylaxis. Therefore, most cases of hydrops fetalis are now thought to be NIHF^2^.

The incidence of NIHF varies from 1 in 1,500 to 1 in 4,000 births^3^. Although most NIHF cases are idiopathic, common causes include cardiovascular anomalies, infectious diseases, and aneuploidies^4^. Prognosis depends on etiology and hydrops subtype, and the perinatal mortality reported in NIHF cases ranges from 50 to 98%^5^. The diagnosis is made by observation of at least two of the following findings: ascites, hydrothorax, pleural effusion, pericardial effusion, and skin edema, defined as 7 mm or more of edema on the fetal scalp^6^. Decisions about the timing and course of delivery in patients diagnosed with NIHF are based on ultrasound findings and prenatal predictive fetal assessments^7^. This study was conducted to compare the gestational age at birth, pregnancy outcomes and associated fetal anomalies, consanguineous parentage status, and prenatal invasive test results in NIHF cases diagnosed before and after 12 weeks of gestation.

## METHODS

**Figure 1:**
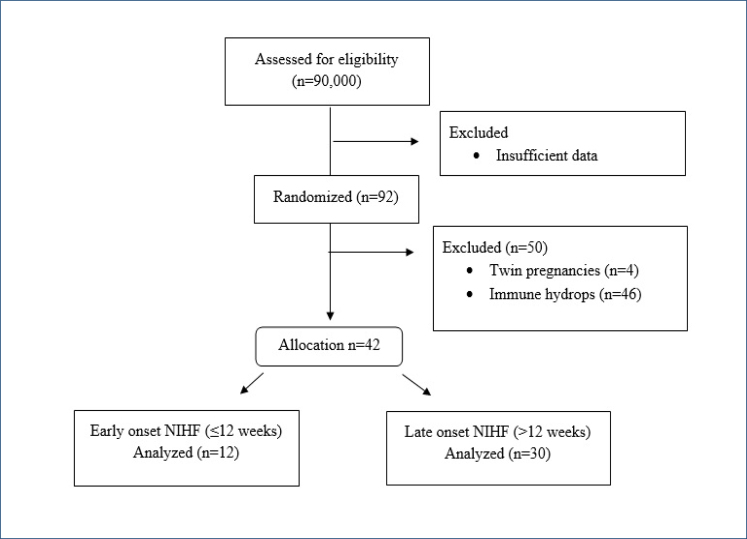
Flowchart of patient inclusion..

This study was designed retrospectively by analyzing patients who applied to the Etlik Zubeyde Hanım Training and Research Hospital between 2017 and 2020 and gave birth. The ethics committee approved the protocol of this hospital study. We adhered to the principles of the Declaration of Helsinki in this study. A total of 90,000 outpatients presenting to our hospital between 2017 and 2020 were studied, and 92 pregnancies with hydrops fetalis in the prenatal period were identified. However, they were excluded from the study because 46 patients had immune hydrops fetalis. Four hydrops patients had multiple pregnancies. Therefore, 42 patients were retrospectively evaluated for etiology (Figure 1). Two clinicians (DŞ and SYE), both experts in fetal anomalies, performed all ultrasound examinations and confirmed the diagnosis of hydrops. For fetal morphology scanning, the Voluson E6 ultrasound system was used in our perinatology clinic (GE Healthcare, Zipf, Austria).

The required pregnancy/delivery data of the patients were obtained from the hospital computer records. Inclusion criteria were singleton pregnancies diagnosed with NIHF during the prenatal period and followed up by our department. NIHFs diagnosed before 12 weeks of gestation were defined as early pregnancy, and NIHFs that began after 12 weeks of gestation were defined as late pregnancy. Exclusion dcriteria for the study population were the presence of multiple pregnancies and immune hydrops fetalis. Gestational age was determined by the last menstrual period or first-trimester ultrasound results. Abortion/week of delivery, birthweight and pregnancy results (termination, intrauterine deaths, neonatal deaths, live births), fetal anomalies, status of consanguineous parents, and prenatal invasive tests as well as array analysis were evaluated. The diagnosis of fetal anemia was excluded by measuring the middle cerebral artery (MCA) peak systolic value and calculating it according to week. Since the cases did not have fetal anemia, a complete blood count was not performed in the neonatal period. In patients diagnosed with NIHF, the timing and course of birth were decided according to ultrasound findings, especially by analyzing fetal heart functions. Families whose babies were born alive were called to confirm defects.

## STATISTICAL ANALYSES

The statistical package SPSS 23.0 IBM was used for statistical analyses. Descriptive statistics included mean, standard deviation, median, frequency, and percentages. Categorical variables were compared using the chi-square test and Fisher's exact test. The normal distribution of continuous variables was assessed using visual (histogram and Q-Q plots) and statistical methods (Kolmogorov-Smirnov tests). In cases where continuous variables were not normally distributed (nonparametric), two groups were compared using the Mann-Whitney U-test. When continuous variables were normally distributed, Student's t-test was performed. The accepted statistical significance level was p<0.05.

## RESULTS

In this study, we found that the prevalence of NIHF in our hospital was 0.05%. In all hydrops groups, the mean age of pregnant women was 28.12±6.55 years, the median gravidity was 2 (range 1–6), and the median parity was 1 (content 0–5). The median week at which hydrops was diagnosed was 18 weeks. Consanguinity (16.7%) was found in seven pregnancies, and the other seven patients (16.7%) had a history of hydrops in previous pregnancies.

In patients diagnosed with NIHF in the first trimester, the diagnosis was confirmed in the second trimester by detailed ultrasonography and invasive test results. We found no statistically significant differences between the two groups with respect to the subgroup of structural abnormalities. Anomalies of the skeletal system, central nervous system, and gastrointestinal tract accounted for 66.7% of ≤12 weeks in NIHF cases. Cardiac abnormalities were more common (26.7%) in patients at>12 weeks (p=0.078). The distribution of these abnormalities by week is shown in Table 1. None of the parents consented to autopsy.

**Table 1:** Comparison of demographic data in the study by the week of nonimmune hydrops fetalis diagnosis.

		Diagnosis week		Total	Test statistics	p
		12 (n=12)	>12 (n=30)			
Age (years)		27.58±6.05	28.33±6.82	28.12±6.55	U=174	0.88
Birthweight (g)		2150.00±1299.7	1799.7±1005.2	1863.4±1038.5	U=28	0.538
NIHF history						
	No	9 (75)	26 (86.7)	35 (83.3)	–	0.387F
	Yes	3 (25)	4 (13.3)	7 (16.7)		
Consanguineous parentage						
	No	9 (75)	26 (86.7)	35 (83.3)	–	0.387F
	Yes	3 (25)	4 (13.3)	7 (16.7)		
Fetal anomalies						
	No	2 (16.7)	9 (30)	11 (26.2)	=8.801	0.185
	Cardiac	1 (8.3)	8 (26.7)	9 (21.4)		
	Skeletal	3 (25)	1 (3.3)	4 (9.5)		
	Central nervous system	3 (25)	3 (10)	6 (14.3)		
	Gastrointestinal system	2 (16.7)	4 (13.3)	6 (14.3)		
	Urinary system	0 (0)	3 (10)	3 (7.1)		
	Pulmonary anomalies	1 (8.3)	2 (6.7)	3 (7.1)		

U: Mann-Whitney U-test statistic; F: Fisher's exact test; : chi-square test statistic; NIHF: nonimmune hydrops fetalis. Results were accepted as 95% confidence interval and p-value <0.05 significant.

The analyses performed revealed no statistically significant difference between NIHF patients detected before 12 weeks of gestation and those detected after 12 weeks of gestation in terms of consanguineous parentage, hydrops history, and fetal anomalies (p=0.387 and =0.185, respectively). The clinical and demographic characteristics of the patients are shown in Table 1.

In our hospital, invasive prenatal screening and array analysis are recommended for all patients diagnosed with NIHF. Cases in which consanguinity was detected and all patients whose prenatal invasive test results were abnormal were referred to genetics. However, only 13 patients (30.9%) agreed to undergo these examinations. Amniocentesis (A/S) was performed in 23.8% (n=10) of these investigations, chorionic villus sampling in 4.8% (n=2), and cordocentesis in 2.4% (n=1). Invasive testing revealed a normal karyotype in nine patients, Trisomy 21 in one patient, Turner syndrome in two patients, and Trisomy 18 in one patient (Table 2). No abnormal results were detected in array analysis.

**Table 2:** Comparison of perinatal outcomes according to diagnosis week of nonimmune hydrops fetalis.

		Diagnosis week		Total	Test statistics	p
		12 (n=12)	>12 (n=30)			
Prenatal invasive test type No		7 (58.3)	22 (73.3)	29 (69)	=5.689	0.128
	A/S	0 (0)	7 (23.3)	10 (23.8)		
	CVS	5 (41.7)	0 (0)	2 (4.8)		
	Cordocentesis	0 (0)	1 (3.3)	1 (2.4)		
Prenatal invasive test results						
	Normal	3 (60)	6 (75)	9 (69.2)	=2.438	0.487
	Trisomy 21	0 (0)	1 (12.5)	1 (7.7)		
	Turner	1 (20)	1 (12.5)	2 (15.4)		
	Trisomy 18	1 (20)	0 (0)	1 (7.7)		
Pregnancy results						
	Termination	3 (27.3)	3 (13)	6 (17.6)	=2.491	0.477
	İntrauterine deaths	5 (45.5)	9 (39.1)	14 (41.2)		
	Neonatal deaths	0 (0)	3 (13)	3 (8.8)		
	Live births	3 (27.3)	8 (34.8)	11 (32.4)		
Delivery type of alive fetuses						
	Vaginal delivery	3 (60)	11 (55)	14 (56)	–	1.000F
	Cesarean section	2 (40)	9 (45)	11 (44)		
Birth week						
	<24	8 (66.7)	7 (23.3)	15 (35.7)	=7.101	0.029
	25–36	1 (8.3)	8 (26.7)	9 (21.4)		
	>37	3 (25)	15 (50)	18 (42.9)		

F: Fisher's exact test; : chi-square test statistic; NIHF: nonimmune hydrops fetalis; A/S: amniocentesis; CVS: chorionic villus sampling. Results were accepted as 95% confidence interval and p-value <0.05 significant.

A statistically significant difference was found between the distribution of the week of birth and the week of diagnosis (p=0.029). Notably, 66.7% of women diagnosed before week 12 and 23.3% of women diagnosed after week 12 delivered before week 24. Meaningful pregnancy data were available in 34 of the 42 patients included in this study. Spontaneous intrauterine death occurred before week 12 in 45.5% (n=5) of NIHFs and after week 12 in 39.1% (n=9) of NIHFs. Overall, 17.6% of pregnancies (n=6) were terminated, 32.4% of babies (n=11) were born alive, and neonatal death occurred in 8.8% of newborns (n=3). When analyzed by weeks of diagnosis, no significant association was found between patients in terms of pregnancy results (p=0.477, Table 2). The prevalence of neonatal mortality in all cases was 25% [(number of infant deaths between 0 and 27 days of life/number of live births) * 1,000] [10]. The delivery method of the live births included in the study is known. Also, 56% of these patients (n=14) delivered their babies vaginally and 44.0% (n=11) by cesarean section (Table 2). Cesarean section was performed in three patients (6.5%) for fetal distress, in five patients (10.8%) for previous uterine surgery, in two patients (4.4%) for abnormal fetal presentation, and in one patient (2.2%) for chorioamnionitis. Intrauterine syphilis infection was detected in one case and cytomegalovirus (CMV) infection in one case. These two cases, detected with maternal blood samples, were also confirmed in the neonatal period. These two patients did not accept prenatal invasive tests. Fetal anemia and parvovirus B19 were not detected in our study population. Maternal complications were not observed during pregnancy and the postpartum period.

## DISCUSSION

This study provides a comparison of the etiology and perinatal outcomes of fetuses diagnosed with NIHF. This is the first study on the etiology and perinatal outcomes of early and late-onset NIHF. The most important finding of this study was that the fetuses diagnosed with early-onset NIHF were also born early, that is, in the first 24 weeks. In addition, live birth rates were higher in fetuses diagnosed with late-stage NIHF.

Early-onset NIHF is mentioned in a limited number of articles in the literature. According to Jauniaux, early hydrops cases were defined between approximately 11 and 14 weeks of age, with no unique week specified^8^. Smeland et al., in their study of a patient with recurrent pregnancy loss due to NIHF at different weeks, defined NIHF as early onset but did not specify a specific week for this definition^9^. Ranganath et al. defined the case of hydrops fetalis beginning at 14 weeks as early-onset hydrops^10^. When analyzing hydrops cases by subdivision after week 12 in our study, we found that the hydrops cases that started in the early weeks, which is an important perinatal outcome, were born earlier than the hydrops cases that started in the later weeks. This allowed us to divide the NIHF cases into early onset and late onset.

In a meta-analysis of 6,361 patients, cardiovascular disease (21.7%) and chromosomal abnormalities (13.4%) were reported as the most common abnormalities in NIHF patients^2^. According to a recent study, the etiology was unknown in 46% (30/65), suspected in 9.2% (6/65), and confirmed in 44.6% (29/65). Of the confirmed cases, 11 resulted from aneuploidy, 7 from fetal structural abnormalities, 2 each from fetal arrhythmias, Noonan syndrome, and generalized lymphocytic dysplasia, and 1 from arthrogryposis, parvovirus, neonatal alloimmune thrombocytopenia, fetal goiter, and Kasabach-Merritt syndrome^11^. In our study, similar to the literature, these were the most common anomaly groups. Although the most common anomaly groups in our study were identical to those in the literature, there was no statistically significant difference between these groups in terms of early- and late-onset NIHF (Table 2). We think that the low rate of prenatal invasive testing in a high-risk population such as NIHF is due to religious reasons. Pediatricians recommended genetic testing to these patients in the neonatal period, but the patients did not undergo it.

According to a retrospective study, the mean week of diagnosis of patients referred with a diagnosis of NIHF was 29.1±4.4, whereas the mean week of delivery of live fetuses was 34.3±2.7^12^. However, all patients included in this study were those diagnosed with hydrops in the third trimester. According to another study, 31.7% of patients diagnosed with NIHF were born before 32 weeks of gestation, 9.4% were born before 28 weeks, and 77.7% of NIHF pregnancies were associated with preterm birth^13^. However, in these two studies, the week in which patients were diagnosed with NIHF was not reported. Notably, 66.7% of NIHF cases with early onset were born before 24 weeks, and 50% of NIHF cases with late onset were born after 37 weeks (Table 2). Our study differs from the literature in this regard.

According to one study, 33.3% of patients diagnosed with NIHF in the previous pregnancy also developed NIHF in the subsequent pregnancy, and 36% of them were diagnosed with lysosomal storage disease^14^. In our study, 16.7% of the patients confirmed that NIHF had complicated their previous pregnancies. Although not statistically significant, the fact that patients with a history of hydrops had more late-stage NIHF than early-stage NIHF could be due to late hospitalization because of problems with previous pregnancies. Future screening of these recurrent NIHF cases for lysosomal storage disorders may be useful.

The consanguinity rate in our country was reported to be 8.4%^15^. Because a high risk of developing autosomal recessive genetic diseases is associated with consanguineous ancestry, screening for NIHF cases is essential. In our study, seven patients (15.2%) had a consanguineous ancestry. We believe that consanguineous ancestry is necessary for the pathophysiology of NIHF and should be questioned.

The prognosis of NIHF depends on the underlying etiology, gestational and birth week, and neonatal status. Even in the absence of chromosomal abnormality, survival rates of less than 50% have been reported in the literature^16,17^. In our study, the neonatal mortality rate was low compared to the literature^18^. However, intrauterine mortality was still higher, supporting the "all or nothing" rule in first-trimester obstetric practice^19^.

Our study has some limitations. First, this is a case–control study with a limited number of patients rather than a prospective study. Most patients did not give consent for invasive prenatal testing, so etiological reasons could not be fully elucidated. It is difficult to confirm the diagnosis of hydrops in fetuses from terminated pregnancies. Because none of the parents consented to autopsy and postpartum genetic testing, we had to limit our diagnosis and confirmation of abnormalities to ultrasonography. In a study of tauopathies in hydrops patients, new-generation rasopathy genes were found in 56% of 26 patients with hydrops fetalis. It has been reported that testing these genes is beneficial in such patients^20^. However, in our country, these genes cannot yet be studied in the perinatal period.

## CONCLUSION

As a result, fetuses diagnosed with NIHF at an early stage may have a more severe course. Although there was no significant difference in etiology between trimesters, cardiac abnormalities were observed to be more common in late-onset NIHF patients. Further studies and new genetic tests are needed to improve treatment and prognosis, better identify ultrasound findings, and perform predictive fetal assessments to justify the indication.

## ETHICS COMMITTEE APPROVAL

The study protocol was approved by the hospital's Medical Research Ethics Department. The authors have confirmed that they have complied with the World Medical Association Declaration of Helsinki regarding the ethical conduct of research involving human subjects (the institutional review board approval number is 14.02.2020/03/16).

## INFORMED CONSENT

Written informed consent for the use of the data was obtained from all persons who participated in this study.
